# Pore Structure as a Response to the Freeze/Thaw Resistance of Mortars

**DOI:** 10.3390/ma12193196

**Published:** 2019-09-29

**Authors:** Ivanka Netinger Grubeša, Berislav Marković, Martina Vračević, Maria Tunkiewicz, Imre Szenti, Ákos Kukovecz

**Affiliations:** 1 Faculty of Civil Engineering and Architecture Osijek, Josip Juraj Strossmayer University of Osijek, Vladimira Preloga 3, 31000 Osijek, Croatia; 2 Department of Chemistry, Josip Juraj Strossmayer University of Osijek, Ulica cara Hadrijana 8/A, 31000 Osijek, Croatia; 3 Civil Engineering Institute of Croatia, Drinska 18, 31000 Osijek, Croatia; martina.vracevic@igh.hr; 4 Institute of Building Engineering, Faculty of Geodesy, Geospatial and Civil Engineering, Heweliusza 4, 10–719 Olsztyn, Poland; maria.tunkiewicz@uwm.edu.pl; 5 Interdisciplinary Excellence Centre, Department of Applied and Environmental Chemistry, University of Szeged, Rerrich Béla tér 1, H-6720 Szeged, Hungary; szentiimre@gmail.com (I.S.); kakos@chem.u-szeged.hu (Á.K.)

**Keywords:** mortar, freeze/thaw resistance, mercury intrusion porosimetry, X-ray micro-computed tomography analysis, SEM analysis

## Abstract

In this paper, the resistance to the freeze/thaw cycles for four groups of mortars (lime—LM, lime based—LBM, cement—CM, and aerated cement—ACM mortars) with different amount of mortar components within each group is quantified via a ratio of flexural/compressive strength after and before exposure to freeze/thaw cycles. Using a pore system obtained by three different methods (mercury intrusion porosimetry, X-ray micro-computed tomography analysis, and SEM (Scanning Electron Microscopy) analysis), an attempt was made to explain why some mortars achieved better resistance to freeze/thaw cycles than others. The mortars with lime as a binder in the composition (LM and LBM groups) did not survive the freezing and thawing regime, while no visible damage was recorded in samples of the CM and ACM group. It is concluded that the low initial value of the mechanical properties of the LM and LBM mortars, as well as the higher proportion of harmful pores (pores greater than 0.064 μm) compared to CM and ACM mortars are responsible for their poor durability. According the results of nanotomography, it is concluded that the most important factor influencing freeze/thaw resistance is pore connectivity—the higher the connectivity of the macropores, the higher the freeze/thaw resistance of the mortar. SEM analysis proved to be a very useful method for aerated cement mortars as it revealed the pore sizes that were not covered by mercury porosimetry and nanotomography.

## 1. Introduction

Some historic buildings, even after many centuries have been passed, are still in good condition today. The reason for this lies in the massiveness of their structures and the large quantities of materials incorporated into such structures, which making them resistant to the aggressive environment and, partly, in the durability of the materials of the past. Today, building preference is given to slender structures with significantly fewer material and, hence, an increased sensitivity to external actions, emphasizing the necessity for material durability [1]. The durability of masonry structures is affected by the strength of the bricks and mortar (the building components), as well as by their mutual compatibility in the wall. Understandably, the durability of mortar increases with increasing resistance towards the action of the degradation agents acting upon it. Such agents may vary considerably, depending on the particular environment (region, climate) where the building is located. In the areas with a cold climate, the effect of the freeze/thaw cycles is the most significant [2]. In a porous material, such as mortar, the freeze/thaw action occurs when water, after entering the micro-cracks through capillary action, freezes. The phase transformation of aqueous solution into solid (ice), in which the volume increases by about 9% [3], leads to the development of high tensile stresses and increase in the crack sizes [4], which causes a loss in the mechanical materials performance [5]. The propagation of micro-cracks through mortar paste, aggregate, and the interfacial transition zone between paste and aggregate is the main factor in the freeze/thaw damages [5,6].

The durability of hardened mortars exposed to freeze/thaw cycles depends on their ability to: resist the water penetration, quickly loose water which would prevent the ice formation inside the mortar, form a porous structure that can withstand the strain caused by the successive cycles of phase transformation and an increase in water volume [7].

A standardized method to estimate the resistance of mortar to freeze/thaw cycles does not exist. Therefore, researchers are applying different protocols to study the freeze/thaw effects on mortar specimens. The authors in study [8] investigated the resistance of mortar to freeze/thaw cycles on the panels made of frost resistant brick applying the procedure that is prescribed in CEN/TS 772-22 [9], which is usually used for brick units. The authors in [10,11] treated their specimens according to RILEM-CDF, the authors in [12,13] applied a regime prescribed in ASTM C666 [14], the authors in [4] conducted tests according to EN 12371, which is applicable to the frost resistance measurements of natural stone, the authors in [15] used CEN/TS 12390-9, which is intended for the frost resistance determination of concretes, and the authors in [7] devised their own regime to simulate the freeze/thaw cycles.

In order to estimate mortar’s resistance to freeze/thaw cycles, researchers have been studying the influence of applied tests on various features and measured values: changes in the surface appearance of samples [8,13], changes in the compressive and flexural strength as well as dynamic modulus of elasticity [12,13,15,16], changes in the weight of the specimens [4,7,12], water absorption [15,16], pore structure and its changes during the cycles [10], and the number of freeze/thaw cycles the specimens can resist until complete disintegration [4,7]. During the freeze/thaw cycles, the surface of the mortar specimens becomes damaged, its compressive and flexural strength (as well as dynamic modulus of elasticity and weight) are decreased, and its water absorption is increased. With each freeze/thaw cycle, new micropores and cracks appeared.

In the case of cement mortars, the ways of improving freeze/thaw resistance are more diverse when compared to lime based mortars. According to [17], the resistance of mortar to freeze/thaw cycles can be enhanced by adding an air entraining agent in the mortar mixture. On the other hand, the enhancement of frost resistance in the case of cement-based composites has been reported by many studies using pozzolanic materials, such as zeolites [18], waste glass sludge [19], ground clay brick [20], calcined clay [16], palm oil clinker powder [21], and nanosized particles [5]. Additionally, it has been shown in several reports that short fibers can be effective as reinforcements [22,23,24]. In the case of lime mortars, cement is known to improve their durability. However, then they are no longer “lime mortars” but “lime based mortars” [25].

Within the term porosity or the pore system, it is important to distinguish gel pores, capillary pores, and macro pores of different sizes [15]. The capillary and macro pores are reported to be the most detrimental for the durability of a mortar [26]. However, different studies list different pore ranges for different pore groups. The authors in [26] claim that pores with a diameter less than 0.01 µm are gel pores, pores from 0.1 to 1 μm are capillary pores, and those larger than 1 μm are macropores. Pores between 0.01 and 0.1 μm are called transitional pores in [26]. The authors in [27], on the other hand, consider gel pores up to 0.0025 µm, capillaries from 0.1 to 10 µm, and macros larger than 10 µm. In [15], the pores are divided into groups according to their size, as follows: gel (up to 0.03 µm), capillary (0.03–10 µm), and macro (over 10 µm). The authors in [6] divided the pores into micro (up to 0.01 µm), meso (0.01–0.05 µm), and macro (0.05–1 µm) pores and cracks (over 1 µm). The authors in [28] classified the pores directly by their effects on the resistance to freeze/thaw cycles: harmless (up to 0.02 µm), less harmful (0.02–0.05 µm), harmful (0.05–0.2 µm), and more harmful (larger than 0.2 µm).

Lime based mortars have been used in building construction for centuries. The appearance of cement as a binder in mortars led to its widespread and excessive use in buildings, including the conservation and rehabilitation of the architectonic heritage. Some of its properties, such as its fast hardening and high mechanical strength, were the determining factors for this overuse. Since the appearance of cement, lime-based mortars have been gradually used less and less frequently. Some decades ago, it was noted that the replacement of lime mortars by cement mortars for the conservation and rehabilitation of old buildings was a serious mistake. Cement mortars are chemically and mechanically incompatible with old building materials because they possess high mechanical properties, rapidly reach maximum strength, have a hardening process that results in the release of soluble salts, and still have a shorter life when compared to lime mortars. After realizing these problems, a new phase began in the use of lime and a new search was undertaken to optimize the behavior of lime mortars. Nevertheless, each of these mortars has an application in a particular type of structure, and, therefore, it is important for them to possess a high durability.

In this paper, the resistance to freeze/thaw cycles of four groups of mortars (lime, lime based, cement, and aerated cement mortars), with different ratios of mortar components within each group, is quantified. Characterizing the pore systems by three different methods (mercury intrusion porosimetry, X-ray micro-computed tomography analysis, and SEM analysis), an attempt was made to explain why some of the mortars achieve better resistance to freeze-thaw cycles than others.

## 2. Experimental Part

### 2.1. Materials and Methods

The mortar mixtures were prepared with a CEM II/B-M (P-S) 32.5R with a density of 3050 kg/m^3^, slaked lime powder CL 80 S with a density of 2240 kg/m^3^, and river sand with a density of 2650 kg/m^3^. The composition of mortar mixtures is shown in Table 1. The designations LBM correspond to lime based mortar, LM to lime mortar, CM to cement mortar, and ACM to aerated cement mortar, while the numbers next to these designations indicate the volume ratio of the cement, lime, and aggregate in the mixture. The air entraining agent Energyair LP K 200 was used in ACM mixtures in the amount of 0.3% of cement mass. All the components were mixed as prescribed in EN 1015-2 [29].

Mortar, in its fresh state, was tested for its consistency according to EN 1015-3 [30], for its density according to EN 1015-6 [31], and for its pore content, according to EN 1015-7 [32]. The results of the mortar testing in its fresh state are given in Table 2. It is evident from Table 2 that within each mortar group, at the same water to binder ratio, the consistency decreases as the density and pore content increases. Namely, the consistency decreases with an increase in the total binder content (cement + lime) within each mortar group, which also increases the binder to sand ratio. The binder contains finer particles than sand; this ultimately results in a lower consistency of mortar mixtures with a higher ratio of fine to coarse particles. At the same time, it is precisely the higher proportion of fine particles in mortar mixtures with a higher ratio of binder to sand that causes these mixtures to have a higher pore content. A higher content of finer particles causes an increase in density as well.

The mortar specimens, prisms with dimensions of 4 cm × 4 cm × 16 cm, were cured in the way prescribed in EN 1015-11 [33]. For the purpose of assessing the effects of the freeze/thaw cycles, the mortar specimens were subjected to compressive and flexural strength tests before and after their exposure to freeze/thaw cycles, and the ratios of these measured properties were calculated. A higher compressive/flexural strength ratio means a better material resistance to freeze/thaw action. To simulate freeze/thaw action, the procedure prescribed by HRN B.D8.011 [34] (the former Croatian regulation for checking brick resistance to freeze/thaw cycles) was used. According to this regulation, the samples saturated with water were placed in the freezer and exposed to a temperature of −20 ± 2 °C for four hours. After that, the samples were immersed in room temperature water where they were kept for four h at a temperature between +15 to +20 °C. This cycle was repeated twenty-five times. The mortar is considered to be resistant to freeze/thaw cycles if the signs of damage (cracks or disintegration) are not visible on any of the tested samples after the described procedure. The compressive and flexural strengths of the mortar specimen were tested according to EN 1015-11 [33]. The tests were performed in triplicate.

The prepared and dried samples that were cut from the mortar specimens prior to the freeze and thaw cycles (cylindrical, 15 mm in diameter and 20 mm high) were tested in a mercury porosimeter. This is a reference test device for mortar porosity testing, because such materials have an incompressible structure. The measuring cycle included mercury intrusion in a pressure range from 2 to 33,000 psi which enables the determination of pores in a range from 800 μm to 0.006 μm. Representative cylindrical specimens of an almost identical volume (about 1.85 cm^3^) were prepared for the measurement. All the samples were cleaned of dust and pollution by compressed air. During the test in the penetrometer (the measuring vessel), the meniscus decreases in the calibrated capillary as the pressure rises. This is the effect of mercury injection into the pores. Using the Washburn equation, the decrease of the mercury level in the capillary is converted into the content of specific pore diameters [35].

In addition, the distribution of pores in the samples was obtained via X-ray micro-computed tomography analysis, which was conducted using X-ray Nanotomography equipment (Bruker Skyscan 2211). The sample dimension of ~2 × 2 × 2 mm^3^ was scanned using a 11 Mpixel cooled CCD camera by applying the source voltage of 100 kV and the source current 400 μA with an exposure time of 300 ms. The voxel size of these datasets was 0.4 μm. The NRecon reconstruction software was used to reconstruct the projected images with a pixel size of 4032 × 2688, and the CTan and CTvol software packages were used to represent the 3D models. The pore range detected in the specimens was 8–100 µm. The open cylindrical volumes of interest (VOIs) used for the quantitative analysis of the reconstructed images were individually optimized for each sample to ensure the optimal representation of the heterogeneous pore structure in the VOI-based calculations.

For the nanotomography, the mortar samples were crushed and sieved on sieve meshes with sizes of 4 and 2 mm, and particles that remained on the 2 mm mesh size were used.

SEM images were taken in a secondary electron imaging mode using a Hitachi S-4700 microscope operating with a cold field emission gun. The acceleration voltage and the magnification were kept constant at 10.0 kV and x40, respectively. Samples were made conductive by sputtering an approx. 10 nm thick gold coating on their surface.

### 2.2. Results and Discussion

The average values of the compressive and flexural strengths of the mortar specimens prior to and after their exposure to freeze/thaw cycles, as well as the changes in each of their properties, are given in Table 3, together with their corresponding standard deviations. The mortars with lime as a binder in their composition (the LM and LBM groups) did not survive the freezing and thawing regime, while no visible damage was observed for the samples in the CM and ACM groups.

From the data listed in Table 3, it can be seen that, prior to the exposure to the freeze/thaw cycles, the LM group of mortars have the worst mechanical properties (compressive and flexural strength), the LBM group achieved slightly better mechanical properties than the LM group, the CM group has the highest mechanical property values, and the ACM group (the only one with the addition of an air entraining agent) achieved slightly worse mechanical performance than the CM group. The same table shows the trend that mortars with a higher mass proportion of binders within the same group (LBM, LM, CM, and ACM) achieved better mechanical properties, e.g., the compressive and flexural strength of LBM 1-2-6 is higher than the compressive and flexural strength of LBM 1-2-9, and the compressive and flexural strength of LBM 1-2-9 is higher than the compressive and flexural strength of LBM 1-2-12. This is not surprising given that the literature also notes higher compressive strengths for mortars with a higher binder content [21] and, to some extent, with a higher lime content [36].

Furthermore, an insight into Table 3 shows that the mortars with lime as a binder in their composition (LM and LBM groups) did not survive the freezing and thawing regime. The reason for this is probably due to the fact that the Ca(OH)_2_, as the main component of the lime mortar, is slightly soluble in water [37] and the applied regime of exposure to freezing and thawing cycles suggests saturation of the samples with water, as well as the fact that these samples achieved low initial mechanical property values. According to [38], the high initial mechanical properties play a significant role in the resistance of cement composites to freezing and thawing cycles. The CM and ACM groups, with their higher values of initial mechanical property values survived the applied test regime. 

Comparing the mechanical properties of the mortars that have survived this freeze/thaw regime, cement and the aerated cement mortars reveals that the addition of an air entraining agent improved the mortar’s resistance, i.e., the compressive and flexural strength ratios after and before the freeze/thaw cycles were better in the aerated cement mortars (the compressive/flexural strength ratio after and before freeze/thaw cycles of ACM 1-4 was higher than the ratio of CM 1-4, the ratio of ACM 1-3 was higher than the ratio of CM 1-3, and the ratio of ACM 1-2 was higher than the ratio of CM 1-2). Again, the mortar mixtures with a higher content of the binder achieved better freeze/thaw resistance in terms of their compressive/flexural strength ratio after and before the freeze/thaw cycles; e.g., the resistance of CM 1-3 was better than the resistance of CM 1-4, while the resistance of CM 1-2 was better than the resistance of CM 1-3. The pore size distribution and total porosity of the mortar samples are given in Table 4.

Analyzing the results presented in Table 4. it can be noticed that the LBM mortar is characterized by the uneven distribution of open pores, and the entire porosity is in the range of 0.25–1.0 μm. Pores of a 0.5 μm diameter are dominant. Total porosity ranges from 32% to 43%. The LM mortar has a similar pore structure, where pores with a diameter of 0.12–3.0 μm, depending on the type of the sample used for the test, are dominant. Porosity of these samples ranges from 33% to 46%. In both types of mortar, there is a single peak with a dominant size.

The other two mortars (CM and ACM) are characterized by a different distribution of pores. Based on the results, it can be concluded that in the CM mortar, most open pores are in the range of 0.032–2 μm. In each of the discussed ranges, the pore sizes presented in the table are of a similar size and oscillate between 10–20%. The total porosity was 28–29%. The last ACM mortar, with increased air content, contains two compartments of dominant porosity. The first, in the range from 0.5 to 2.0 μm (more than half of the obtained porosity), and the second range, from 0.064 to 0.5 μm, which includes about 30% of all pores. Total porosity in the ACM mortars was 30–35.6%.

**Figure 1:**
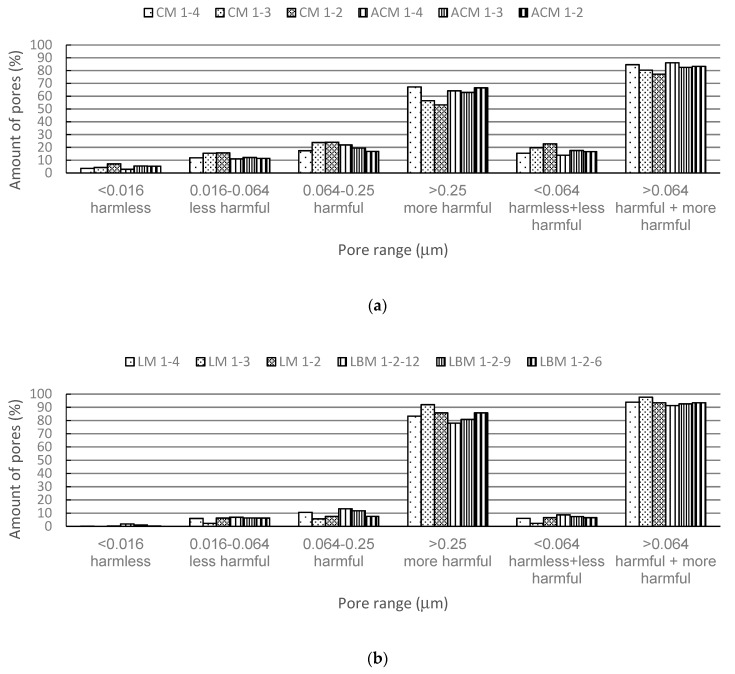
Pore groups with respect to their harmfulness (**a**) in cement and aerated cement mortars and (**b**) in lime and lime based mortars.

Furthermore, the pore ranges given in Table 4 are grouped into four main groups: pores with a diameter up to 0.016 μm, 0.016–0.064 μm, 0.064–0.25 μm, and pores with a diameter of 0.25 μm and greater, which are shown in Figure 1a,b. Namely, such a grouping classifies pores as harmless, less harmful, harmful, and more harmful according to [28], whereby the pore grouping is adapted to the pore ranges recorded in Table 4.

Figure 1a,b shows that the cement and aerated mortars developed higher level of harmless and less harmful pores, as well as lower levels of harmful and more harmful pores then the lime and lime based mortars which could be one of the reasons that justifies the lower resistance of lime and lime based mortars to freeze/thaw cycles. For the cement and aerated cement mortars, it is evident that within their own mortar group, mortars with a higher proportion of binder have a smaller proportion of harmful and more harmful pores, which is accurately matched to the ratios of the mechanical properties after and before the freeze/thaw cycles for the mortars shown in Table 3. This result, again, evidences the detrimental effect of macro pores (larger than 0.064 μm) on the freeze/thaw resistance of mortar.

The quantity of open and closed pores, as well as their connectivity, was obtained by nanotomography (Table 5). 

It can be observed in Table 5 that there are discrepancies in the content of pores with diameters 8–100 µm obtained by nanotomography, and the sum of the pores ranges from 8 μm upwards (8–16, 16–32, 32–… µm) (as shown in Table 4). Discrepancies between the mercury porosimetry and nanotomography derived porosity data are due to (i) the fundamental differences between the operating principles of the two methods and (ii) the lack of a complete overlap between the pore diameter range measurable by the techniques. Even so, the qualitative agreement of the trends observed by these fully independent methods provides strong evidence for the validity of the argument presented here. Total porosity (closed + open) and open porosity are clearly much higher for the CM and ACM samples than for the LM and LBM ones. Considering the fact that this method recorded pores in a range from 8 to 100 μm (which were classified in the previously mentioned literature as harmful), it was expected that such a high content of macropores would have a detrimental effect on the freeze/thaw resistance of the CM and ACM mortars. In addition, the authors in [39] observed that the materials with the highest proportion of open pores, as in the case of CM and ACM mortars, are the least resistant to freeze/thaw cycles. On the other hand, CM and ACM mortars showed superior freeze/thaw resistance over LM and LBM mortars (Table 3). At the same time, the pore connectivity recorded by nanotomography (Table 5) was much higher in CM and ACM mortars than in LM and LBM, leading to the conclusion that pore connectivity is a very important factor for the freeze/thaw resistance of mortar—the higher the connectivity of the macropores, the higher the freeze/thaw resistance of the mortar. Namely, the assumption is that water, after entering the mortar once, can leave the mortar structure faster in the case of higher pore connectivity, thereby diminishing the likelihood of water retention and damaging the mortar structure caused by freezing.

**Figure 2:**
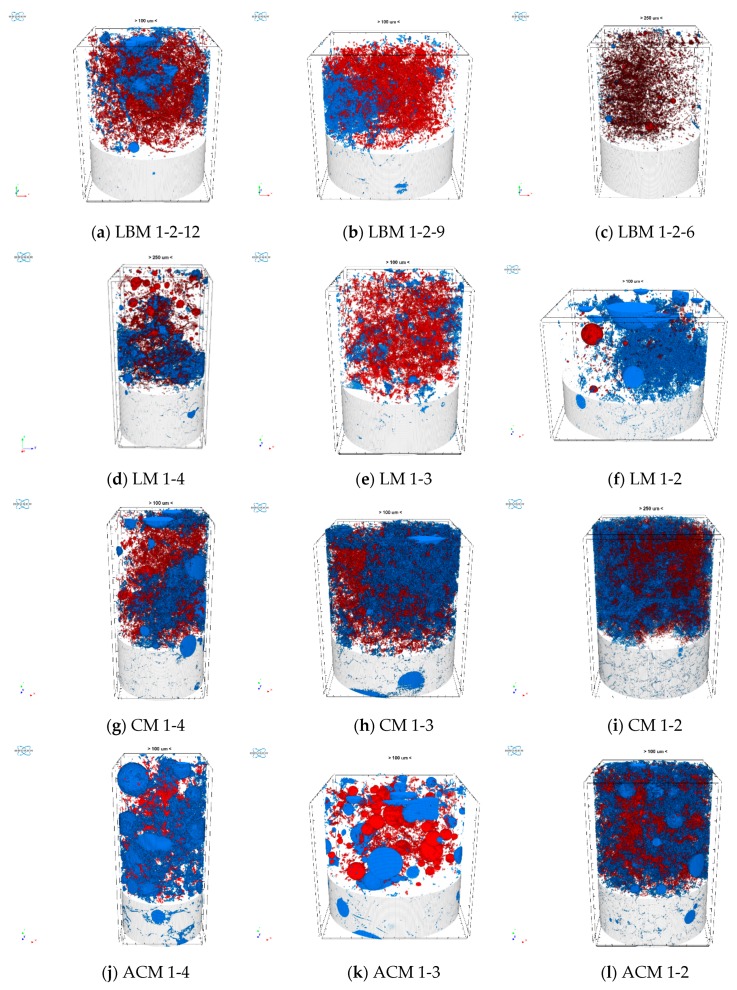
Nanotomography images of the mortar samples: red color-closed pores and blue color-open pores. (**a**) sample of mortar type LBM 1-2-12; (**b**) sample of mortar type LBM 1-2-9; (**c**) sample of mortar type LBM 1-2-6; (**d**) sample of mortar type LM 1-4; (**e**) sample of mortar type LM 1-3; (**f**) sample of mortar type LM 1-2; (**g**) sample of mortar type CM 1-4; (**h**) sample of mortar type CM 1-3; (**i**) sample of mortar type CM 1-2; (**j**) sample of mortar type ACM 1-4; (**k**) sample of mortar type ACM 1-3; (**l**) sample of mortar type ACM 1-2.

Further, the open cylindrical volumes of interest (VOIs) used for quantitative analysis of the reconstructed images were individually optimized for each sample to ensure the optimal representation of the heterogeneous pore structure in the VOI-based calculations as shown in Figure 2. Closed pores are marked here in a red color, the open pores are marked in a blue color, and the matrix is colored in grey. 

In Figure 2, one can visually see what was previously shown numerically in Table 5., i.e., that closed pores (red color) prevail in LBM mortars, while in all the other mortars (with the exception of LM 1-3), open pores (blue color) are dominant.

**Figure 3:**
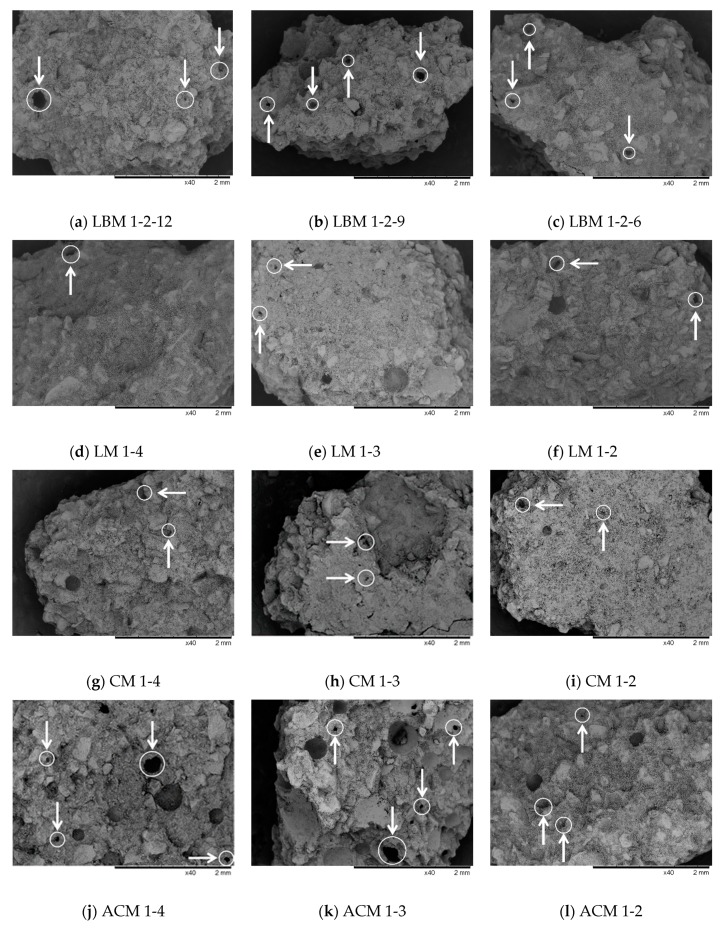
Characteristic SEM images of mortar samples recorded at ×40 magnification. (**a**) sample of mortar type LBM 1-2-12; (**b**) sample of mortar type LBM 1-2-9; (**c**) sample of mortar type LBM 1-2-6; (**d**) sample of mortar type LM 1-4; (**e**) sample of mortar type LM 1-3; (**f**) sample of mortar type LM 1-2; (**g**) sample of mortar type CM 1-4; (**h**) sample of mortar type CM 1-3; (**i**) sample of mortar type CM 1-2; (**j**) sample of mortar type ACM 1-4; (**k**) sample of mortar type ACM 1-3; (**l**) sample of mortar type ACM 1-2.

The results of the SEM analysis are shown in Figure 3. 

In Figure 3, it can be seen that a few of the pores with sizes of 100–300 μm in the LBM mortars, a few pores of 100–200 μm in the CM mortars, and some cavities in the LM 1-3 and LM 1-2 mortars likely resulted from insufficient compaction of the samples, as well as the large number of pores sized 100–500 μm in the ACM mortars. These pore diameter ranges are markedly different from those observed either by mercury porosimetry or by microCT analysis. Therefore, it is safe to conclude that even though the intrinsic limitations of scanning electron microscopy only allow us to see open pores, this method identified a third pore diameter range. These pores (air voids) caused by the use of an air-entraining agent (the ACM group of mortars) were likely formed and evenly distributed. After freeze/thaw cycles, these air voids helped to ease volumetric expansion and cut off potential water channels to improve the frost resistance of the cement composite [27]. According to Zhang [27], in the case of two mortar mixtures with the same air content, the one with a smaller pore diameter, a greater number of pores, and a smaller air void spacing factor will possess better frost resistance. Almost the same air content for mortar of ACM group is visible in Table 4, while a smaller size and greater number of pores are visible in Figure 3 for ACM 1-2 compared to ACM 1-4. This visual observation is very well matched to the ratios of the mechanical properties before and after the freeze/thaw cycles for the mortars shown in Table 3, i.e., the ratio of the mechanical properties before and after the freeze/thaw cycles were higher fir ACM 1-4 than for ACM 1-2.

## 3. Conclusions

The resistance of the four groups of mortars (lime-LM, lime based-LBM, cement-CM, and aerated cement-ACM mortars) to freeze/thaw cycles, with different amounts of mortar components within each group, was quantified. The mortars with a higher mass proportion of binders within the same group of mortars (groups LBM, LM, CM, and ACM) achieved better initial mechanical properties. For freeze/thaw resistance, the mortars with lime as a binder in the composition (LM and LBM groups) did not survive the freezing and thawing regime, while no visible damage was recorded on the samples of the CM and ACM group. Examining the pore system via three different methods (mercury intrusion porosimetry, X-ray micro-computed tomography analysis and SEM analysis), the authorstried to clarify the freeze/thaw testing results. It can be concluded that a low initial value for the mechanical properties of LM and LBM mortars, as well as the higher proportion of harmful pores (pores greater than 0.064 μm) in comparison to the CM and ACM mortars, are responsible for their poor durability. However, within the CM and ACM groups, some mortars were found to have better resistance than others. According to the results of nanotomography, it can be concluded that the most important factor influencing freeze/thaw resistance is pore connectivity—the higher the connectivity of the macropores, the higher the freeze/thaw resistance of the mortar. SEM analysis proved to be a very useful method for studying the aerated cement mortars, as it revealed pores in a size range that was not covered by mercury porosimetry and nanotomography.

## Figures and Tables

**Table 1:** The composition of mortar mixtures.

Mortar Type/Constituent	LBM 1-2-12	LBM 1-2-9	LBM 1-2-6	LM 1-4	LM 1-3	LM 1-2	CM 1-4	CM 1-3	CM 1-2	ACM 1-4	ACM 1-3	ACM 1-2
Cement (kg)	149.73	175.58	212.23	-	-	-	459.95	541.63	658.59	459.95	541.63	658.59
Lime (kg)	219.92	257.90	311.74	328.58	385.06	465.0	-	-	-	-	-	-
Water (kg)	263.67	309.20	373.75	266.57	312.40	377.25	245.98	289.67	352.21	245.98	289.67	352.21
Water/binder	0.71	0.71	0.71	0.81	0.81	0.81	0.54	0.54	0.54	0.54	0.54	0.54
Air entraining agent (kg)	-	-	-	-	-	-	-	-	-	1.38	1.63	1.98
Sand (kg)	1561.03	1372.97	1106.38	1554.87	1366.61	1100.20	1598.52	1411.79	1144.43	1598.52	1411.79	1144.43

**Table 2:** Properties of mortar in its fresh state.

Mortar Type/Property	LBM 1-2-12	LBM 1-2-9	LBM 1-2-6	LM 1-4	LM 1-3	LM 1-2	CM 1-4	CM 1-3	CM 1-2	ACM 1-4	ACM 1-3	ACM 1-2
Consistency (cm)	210	160	130	210	170	135	210	140	105	210	170	110
Density (g/cm^3^)	1.55	1.67	1.74	1.42	1.50	1.66	1.86	1.87	1.92	1.79	1.83	1.85
Pore content (%)	0.6	1.7	4.8	2	1.5	4.5	1.3	3.3	6.9	4.3	9.6	12

**Table 3:** Properties of mortar specimens prior and after their exposure to freeze/thaw cycles with the corresponding standard deviations.

Mortar Type/Property	LBM 1-2-12	LBM 1-2-9	LBM 1-2-6	LM 1-4	LM 1-3	LM 1-2	CM 1-4	CM 1-3	CM 1-2	ACM 1-4	ACM 1-3	ACM 1-2
Flexural strength (MPa)	1.2 ± 0.1	1.3 ± 0.1	1.6 ± 0.1	1.0 ± 0.1	1.1 ± 0.1	1.3 ± 0.1	6.1 ± 0.4	6.3 ± 0.4	6.4 ± 0.5	4.7 ± 0.4	4.9 ± 0.4	5.6 ± 0.5
Compressive strength (MPa)	4.0 ± 0.2	4.7 ± 0.2	5.2 ± 0.3	1.8 ± 0.1	2.3 ± 0.2	2.6 ± 0.2	30.3 ± 2.1	30.9 ± 2.4	31.3 ± 2.9	26.8 ± 2.1	27.2 ± 1.9	28.3 ± 2.3
Flexural strength after exposure to freeze/thaw cycles (MPa)	-	-	-	-	-	-	4.7 ± 0.4	5.0 ± 0.4	5.3 ± 0.5	4.1 ± 0.3	4.2 ± 0.4	5.1 ± 0.5
Compressive strength after exposure to freeze/thaw cycles (MPa)	-	-	-	-	-	-	23.1 ± 1.9	23.8 ± 2.0	26.8 ± 2.3	22.5 ± 2.3	23.4 ± 2.1	26.3 ± 2.1
Ratio of flexural strength after and before exposure to freeze/thaw cycles	-	-	-	-	-	-	0.77	0.79	0.83	0.87	0.86	0.91
Ratio of compressive strength after and before exposure to freeze/thaw cycles	-	-	-	-	-	-	0.76	0.77	0.86	0.84	0.86	0.93

**Table 4:** Pore size distribution and total porosity of mortar specimens.

Sample/Amount of Pores of a Specific Size (%)	Pore Diameter Range (µm)											Total Porosity (%)				
	−32	32–16	16–8	8–4	4–3	3–2	2–1	1–0.5	0.5–0.25	0.25–0.12	0.12–0.064	0.064–0.032	0.032–0.016	0.016–0.008	0.008–0.006	-
LBM 1-2-12	2.0	0.2	0.2	0.2	0.2	0.5	10.9	39.7	24.1	9.0	4.3	4.0	2.9	1.4	0.4	32.4
LBM 1-2-9	2.2	0.1	0.1	0.1	0.0	0.3	11.3	48.2	18.5	7.4	4.4	3.9	2.5	0.9	0.1	36.2
LBM 1-2-6	3.4	0.1	0.1	0.1	0.1	0.4	15.4	45.7	15.5	7.5	4.6	4.3	2.1	0.6	0.1	43.8
LM 0-1-4	2.4	0.1	0.2	0.6	2.0	5.4	10.9	40.5	21.2	6.9	3.7	3.8	2.2	0.1	0.0	33.7
LM 0-1-3	2.1	0.2	0.2	0.2	0.7	20.9	52.5	10.4	4.8	2.7	3.0	2.0	0.3	0.0	0.0	40.6
LM 0-1-2	1.9	0.1	0.1	0.1	0.0	0.1	17.6	56.0	9.9	4.7	2.9	3.9	2.4	0.3	0.0	46.1
CM 0-1-4	4.3	0.2	0.5	3.1	5.9	14.6	15.1	11.3	12.2	10.2	7.2	6.3	5.5	3.2	0.4	28.9
CM 0-1-3	2.1	0.1	0.2	0.2	0.4	3.0	21.8	13.6	15.1	13.3	10.5	8.6	6.8	3.8	0.5	28.7
CM 0-1-2	3.7	0.1	0.2	0.3	0.2	0.6	10.2	23.5	14.4	13.7	10.3	8.6	7.1	6.0	1.1	29.0
ACM 0-1-4	8.1	0.5	0.6	1.8	1.8	7.5	22.9	11.2	9.8	11.1	10.8	7.1	3.9	2.3	0.6	30.1
ACM 0-1-3	3.7	0.4	0.3	0.3	0.6	2.7	31.4	16.2	7.4	9.8	9.7	7.1	5.0	4.5	0.9	33.6
ACM 0-1-2	4.6	0.5	0.6	0.9	1.0	5.6	37.3	8.3	7.7	8.9	7.9	6.2	5.2	4.7	0.6	35.6

**Table 5:** The amount of open and closed pores, as well as their connectivity obtained by nanotomography.

Mortar Type/Pore Content	LBM 1-2-12	LBM 1-2-9	LBM 1-2-6	LM 1-4	LM 1-3	LM 1-2	CM 1-4	CM 1-3	CM 1-2	ACM 1-4	ACM 1-3	ACM 1-2
Closed (%)	0.99	1.40	0.46	0.93	0.85	1.62	1.75	1.25	1.83	0.63	3.7	1.48
Open (%)	0.04	0.97	0.06	2.86	0.37	2.81	3.88	3.55	3.34	11.27	4.96	5.49
Total (%)	1.03	2.38	0.52	3.79	1.21	4.43	5.64	4.79	5.17	12.0	8.66	6.97
Pore connectivity (1/mm^3^)	1432	3749	299	2374	1474	4746	2832	6235	7521	5159	775	1160
